# Supplementary material for: Identification of photoperiod-induced specific miRNAs in the adrenal glands of Sunite sheep (Ovis aries)
Source: Front Vet Sci. 2022 Jul 22;9:888207. doi: 10.3389/fvets.2022.888207 (PMC9354845; doi:10.3389/fvets.2022.888207)
Supplement: Supplementary file 1 [file Data_Sheet_1.ZIP › Supplementary file/miRDeep2 file.zip!/miRDeep2 file/Know_Structure/SPLP42A3/oar-mir-22.pdf]

miRBase precursor : oar-mir-22
Total read count : 60255
oar-miR-22-3p read count : 54212
remaining reads : 6043

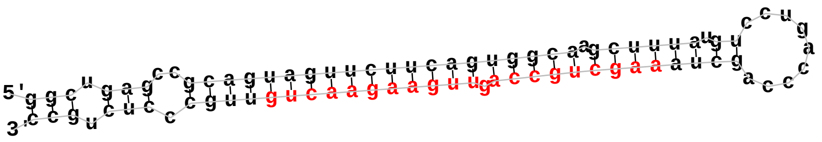

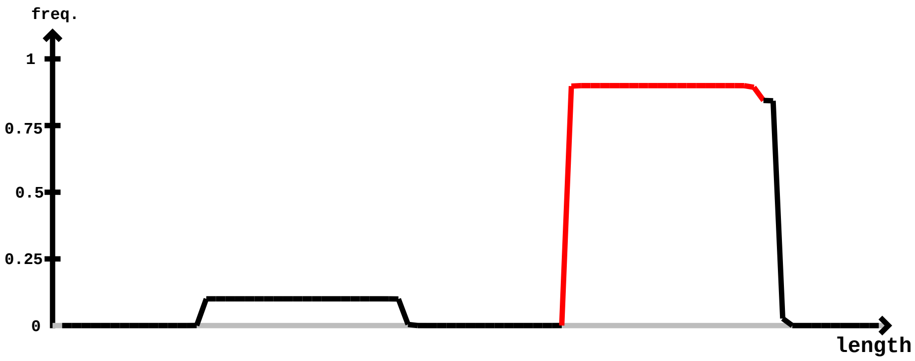

oar-miR-22-3p

```
5'- ggcugagccgcaguaguucuucaguggcaagcuuuauguccugacccagcuaaagcugccaguugaagaacuguugcccucugcc   -3'   exp
    (((.(((..((((((((((((((((((((.((((((.((.........)))))))))))).)))))))))))))))).))).)))   reads   mm   sample
    .............uaguucuucaguggcaagcuuu..................................................   16      0    seq
    ..............aguucuucaguggcaagc.....................................................   1       0    seq
    ..............aguucuucaguggcaagcu....................................................   2       0    seq
    ..............aguucuucaguggcaagcuu...................................................   30      0    seq
    ..............aguucuucaguggcaagcuuu..................................................   5733    0    seq
    ..............aguucuucaguggcaagcuuua.................................................   252     0    seq
    ..............aguucuucaguggcaagcuuuau................................................   4       0    seq
    ...............guucuucaguggcaagcuuu..................................................   3       0    seq
    ...............guucuucaguggcaagcuuua.................................................   1       0    seq
    .................................................cuaaagcugccaguugaagaacu.............   1       0    seq
    ...................................................aaagcugccaguugaagaacu.............   7       0    seq
    ...................................................aaagcugccaguugaagaacugu...........   4       0    seq
    ....................................................aagcugccaguugaaga................   3       0    seq
    ....................................................aagcugccaguugaagaa...............   12      0    seq
    ....................................................aagcugccaguugaagaac..............   367     0    seq
    ....................................................aagcugccaguuCaagaacu.............   1       1    seq
    ....................................................aagcugccaguugaagaacu.............   2954    0    seq
    ....................................................aagcugccaguugaagaacug............   82      0    seq
    ....................................................aagcugccaguugaagaacugu...........   49092   0    seq
    ....................................................aagcugccaguugaagaacuguu..........   1577    0    seq
    ....................................................aagcugccaguugaagaacuguug.........   1       0    seq
    .....................................................agcugccaguugaagaacu.............   3       0    seq
    .....................................................agcugccaguugaagaacugu...........   91      0    seq
    .....................................................agcugccaguugaagaacuguu..........   17      0    seq
    ......................................................gcugccaguugaagaacugu...........   1       0    seq
```
